# Anticancer drugs targeting topoisomerase II for antifungal treatment

**DOI:** 10.1038/s41598-025-93863-z

**Published:** 2025-03-18

**Authors:** Kavya Kondaka, Kamila Rząd, Natalia Maciejewska, Iwona Gabriel

**Affiliations:** Department of Pharmaceutical Technology and Biochemistry, Faculty of Chemistry, Gdansk University of Technology, 11/12 Narutowicza Str, Gdansk, 80-233 Poland

**Keywords:** Biochemistry, Drug discovery, Microbiology

## Abstract

Fungal topoisomerase II (TopoII) has been identified as essential for viability. Thus, our research aimed to investigate the potential of fungal TopoII as a novel target for antifungal chemotherapy. We conducted studies on eleventh antitumor compounds targeting human topoisomerase II, either approved by the U.S. Food and Drug Administration (FDA) or currently under clinical trials to evaluate their potential for use in other therapeutic applications. While most of the compounds we analyzed are potent inhibitors of yeast TopoII, only a few exhibited antifungal activity. Idarubicin emerged as the most potent compound effectively inhibiting the growth of five reference fungal strains as well as clinical *Candida glabrata* fluconazole-resistant cells. Antifungal activity of this compound corresponded with its very high yeast TopoII inhibitory effectiveness. Additionally, idarubicin ability to be effectively accumulated into fungal cells is crucial for yeast TopoII targeting. Idarubicin, epirubicin, and bisantrene appeared to be even more effective inhibitors of yeast enzyme than its human counterpart. In fungal cells idarubicin exhibited a multifaceted mechanisms of action, including nuclear DNA fragmentation, disruption of mitochondrial network architecture and mitochondrial DNA aggregation as well as oxidative stress induction. Our results indicate that fungal topoisomerase II targeting is worth considering in antifungal treatment and the reported drugs may serve as a starting point for the reinnovation of a new molecule.

**Supplementary Information:**

The online version contains supplementary material available at 10.1038/s41598-025-93863-z.

## Introduction

Fungal microorganisms are etiological agents responsible for a multitude of severe and often fatal infectious diseases, particularly among immunocompromised individuals^1^. This issue is further exacerbated by the increasing prevalence of immunocompromised patients, stemming not only due to conditions such as acquired immunodeficiency syndrome (AIDS) but also as a result of the widespread use of therapies that adversely affect the human immune defense system^2^. Moreover, invasive fungal mycoses have also been associated with COVID-19 patients and survivors^3,4^. In 2022, the World Health Organization launched the first fungal priority pathogens list, categorizing *Candida albicans* together with *Candida auris*,* Cryptococcus neoformans*, and *Aspergillus fumigatus* into the critical threat group of fungal pathogens. These pathogens are considered the most dangerous for public health as they are associated with the highest incidence and mortality^5^. The most often used classes of antifungal drugs target the components of fungal cell wall, membrane, or the biosynthesis of pyrimidine; these groups are echinocandins, azoles, polyenes, and pyrimidines. Apart from being effective, available antifungal therapies are often associated with adverse effects, drug-drug interactions, and prolonged therapy^6^. Thus, despite the increasing number of reports of advances in antifungal therapy, the development of new antifungal drugs seems insufficient in comparison with bacterial research^7^. Mostly, studies are focused on the improvement of existing antifungal drug classes. However, some candidates exhibit novel mechanisms of action, such as inhibition of heat shock protein 90 or calcineurin signaling^7^, the biosynthesis of fungal amino acids, proteins, DNA, and other essential molecules targeting^8–11^.

DNA topoisomerases are indispensable and ubiquitous enzymes, playing a fundamental role in the intricate control and regulation of DNA topology during a plethora of vital cellular processes. These enzymes serve as targets for various antibacterial and anticancer agents^12,13^. Fungal DNA topoisomerases remain underexplored, although topoisomerase II from *Saccharomyces cerevisiae* and *Schizosaccharomyces pombe* was identified as essential for viability^14,15^. TopoII plays critical roles, including relaxation of positive and negative supercoils, decatenation, and DNA unknotting, all in an ATP and Mg^+ 2^ dependent manner^14–17^. In budding yeast (*S. cerevisiae*), TopoII is also responsible for regulating the expression of specific gene subsets. Genes directly regulated by TopoII inactivation in *S. cerevisiae* fall into two subsets: an upregulated subset containing genes poor in essential functions but enriched in TATA-containing genes, responsible for membrane transport of polyamines, and a downregulated subset consisting of essential genes involved in chromatin remodeling and transcriptional regulation^18,19^.

Inhibition studies performed for fungal topoisomerase II also indicated the possibility of targeting that enzyme for new antifungals development^20–24^. Anticancer compounds such as amsacrine (m-AMSA) and etoposide exhibit differential inhibitory properties against fungal and human enzymes. Intriguingly, *Candida* topoisomerases have been found to exhibit reduced sensitivity to m-AMSA and etoposide in comparison to their mammalian counterparts^25,26^. ICRF-187 (dexrazoxane), a catalytic inhibitor of ATPase activity, affects both fungal and human enzymes with significantly higher activity against human TopoII isoforms (hTopoIIα and hTopoIIβ)^27^. Remarkably, these sensitivity variations do not align with the mechanism of action, as both m-AMSA and etoposide function as topoisomerase “poisons” while dexrazoxane acts as a catalytic inhibitor.

From literature, among the clinically used anticancer drugs targeting human TopoII only three display moderate antifungal activity against some fungal strains: etoposide^28,29^, aclarubicin^30^, and idarubicin^30–32^. Aclarubicin exhibits fungistatic activity while daunorubicin, doxorubicin, idarubicin, and β lapachone, influence *C. albicans* morphology^30^. Among compounds being analyzed in clinical trials as antitumor, targeting topoisomerase II, only quinacrine was analyzed and acts against *Candida albicans* cells by inhibiting yeast-to-mycelia transformation and biofilm formation^33^.

**Fig. 1:**
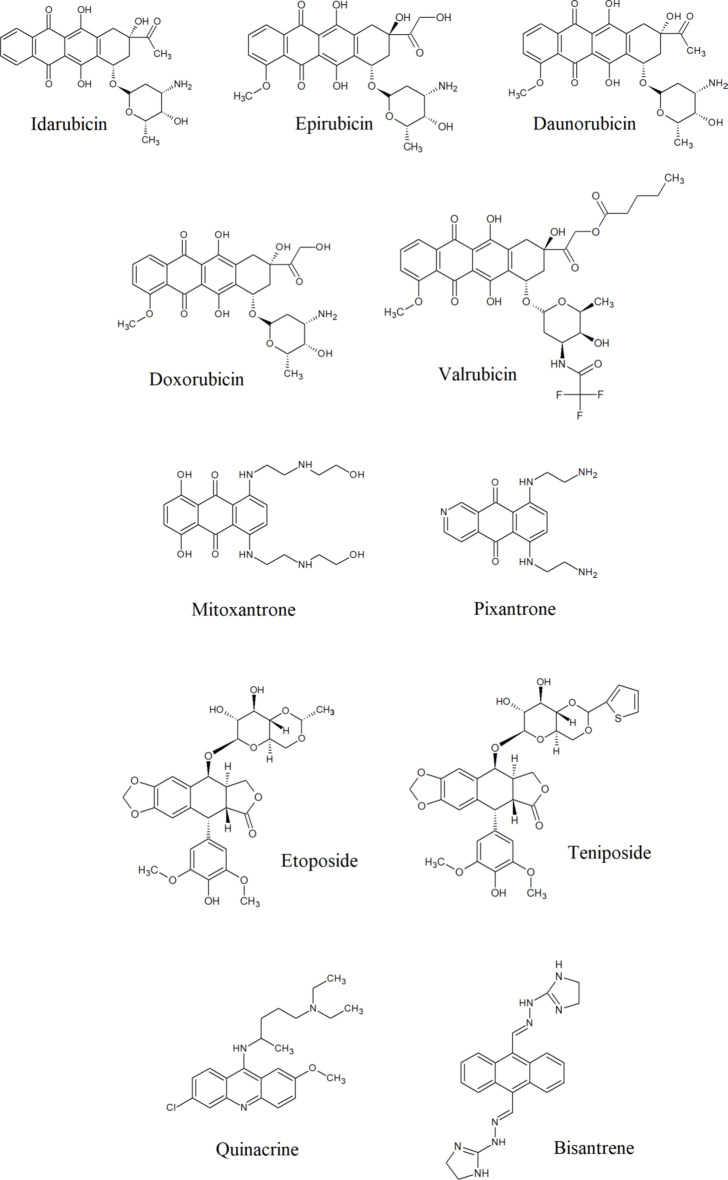
Known human topoisomerase II inhibitors analyzed in this study.

As current methods for preventing fungal infections prove insufficient and due to the emergence of drug-resistant fungal strains infections, we have decided to perform studies among a group of eleventh FDA-approved or being analyzed in clinical trials antitumor compounds, targeting hTopoIIα (Fig. 1), to verify the possibility of being used as drugs in other indications. Such approach known as drug repurposing is considered as a rapid, cost-effective, and reduced-risk strategy for the development of new treatment options. Our results indicated the possibility of using selected compounds as antifungals even against drug-resistant strains.

## Results and discussion

The development of new antifungal drugs represents a major challenge for the pharmaceutical industry since fungi are eukaryotic organisms and have a close evolutionary relationship with their human hosts^34^. On the other hand, previously published preliminary studies performed for selected, FDA-accepted, human topoisomerase II inhibitors as antifungals^28–32^ as well as our promising results on fungal TopoII targeting^21–24^ prompted us to perform more detailed studies. For this purpose we have chosen a group of eleventh antitumor compounds, targeting hTopoIIα (Fig. 1). Eight of them are FDA-approved anticancer drugs and well-known human topoisomerase II inhibitors (doxorubicin, daunorubicin, epirubicin, idarubicin, mitoxantrone, pixantrone, etoposide, teniposide)^35^. One anthracycline derivative: valrubicin (also FDA-approved anticancer drug) is supposed to exhibit increased lipophilicity and rapid cellular penetration ability^36,37^. This effect is contributed to derivatization of the charged amino group with trifluoroacetyl moiety at C-3’and the addition of alkyl ester at C-14 (Fig. 1). This valerate moiety, modifies the pattern of subcellular localization of that compound, leading to a different mechanism of action, not directly related to human TopoII inhibition^36,37^. On the other hand, the valerate-free metabolite of valrubicin is a nuclear-targeted topoisomerase II poison^38^. Two other compounds: quinacrine and bisantrene were analyzed in clinical trials as antitumors also targeting human topoisomerase II^39,40^.

### Inhibition of yeast and human topoisomerase II activity in vitro

**Fig. 2:**
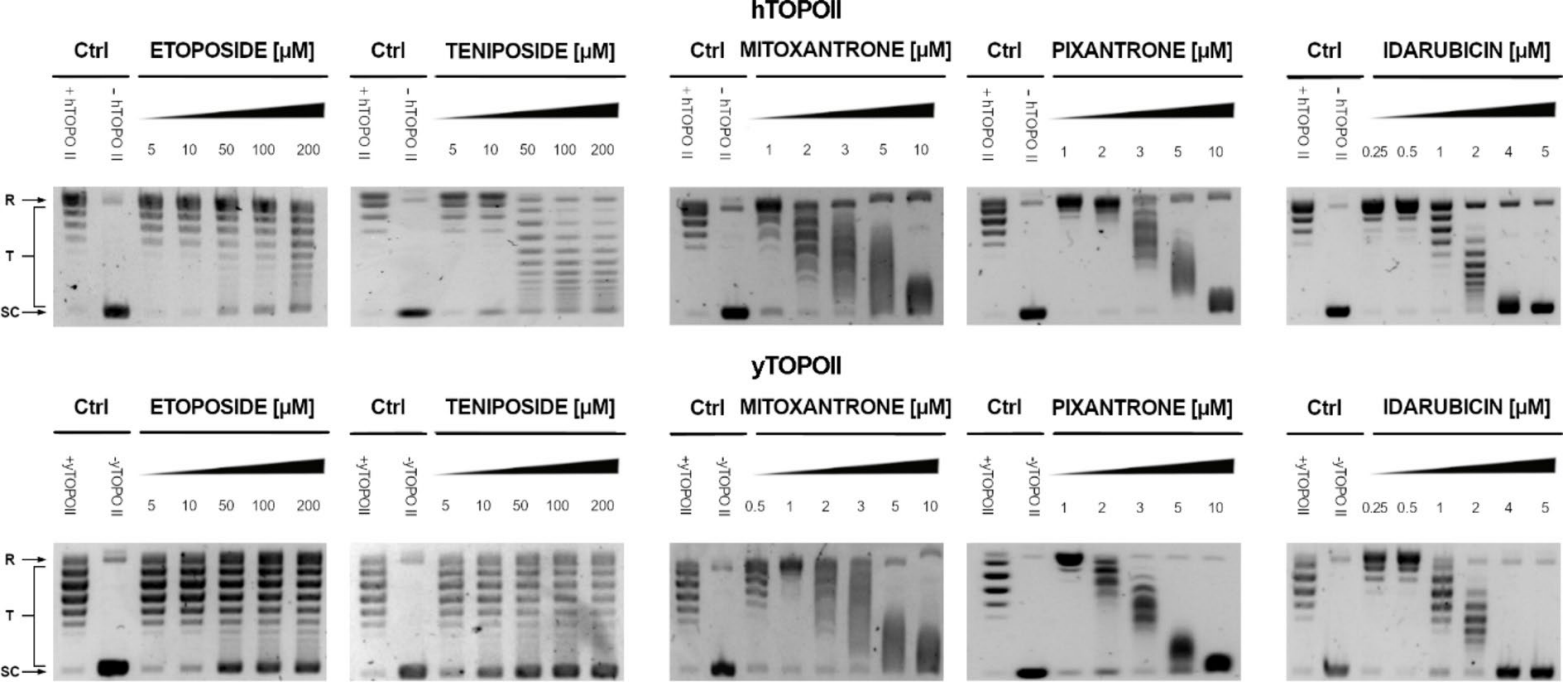
Inhibition of the catalytic activity of purified yeast (yTOPOII) and human (hTOPOII) DNA topoisomerase II by etoposide, teniposide, mitoxantrone, pixantrone, and idarubicin measured by relaxation. Supercoiled pBR322 plasmid DNA (- hTOPO II or –yTOPO II) was relaxed by purified topoisomerase II in the absence (+ hTOPO II or + yTOPO II) or presence of compounds at selected concentrations [µM]. The resulting topological forms of DNA were separated by gel electrophoresis. SC, supercoiled DNA; R, relaxed DNA; T, DNA topoisomers. The data shown are typical of three independent experiments. The gels were cropped to increase the clarity of the presentation. The unedited gels are shown in Supplementary Materials Figure S1 and Figure S2.

We analyzed the effects of all compounds on yeast topoisomerase II (from *S. cerevisiae*) relaxation activity in comparison with inhibitory effectiveness against human enzyme counterpart. Selected results are presented in Fig. 2. The relaxation of supercoiled plasmid DNA by both enzymes was studied in the presence of different concentrations of compounds with the use of a gel-based assay that allow to determine IC_50_ (Table 1).

**Table 1:** The Inhibition activity of the analyzed derivatives determined by densitometry quantification of the transition from supercoiled to relaxed forms. The half maximal inhibitory concentration (IC_50_) refers to the concentration of a drug, which inhibited the relaxation at 50%. The experiments were performed at least in three replicates.

Compound	IC_50_ µM	
	yTopoII	hTopoIIα
Idarubicin	1.298 ± 0.302	2.312 ± 0.321
Epirubicin	1.765 ± 0.067	2.005 ± 0.189
Daunorubicin	1.352 ± 0.446	1.323 ± 0.321
Doxorubicin	15.287 ± 0.984	16.382 ± 0.945
Quinacrine	16.059 ± 0.787	12.267 ± 1.702
Bisantrene	1.596 ± 0.096	2.107 ± 0.095

Our results indicated that the most effective inhibitors appeared to derived from anthracyclines with the most active daunorubicin and idarubicin against fungal enzyme. Moreover, inhibitory effectiveness seemed to be even better for enzyme derived from *S. cerevisiae* than its human counterpart for idarubicin, epirubicin, and bisantrene (Table 1). For pixantrone and mitoxantrone it was not possible to determine IC_50_ due to their strong binding ability to DNA^41,42^ and related to that DNA smearing on the gels (Fig. 2). Regardless, the inhibitory effectiveness appeared to be comparable.

### Susceptibility testing against fungal strains

Known anticancer compounds targeting human TopoII were tested for their in vitro antifungal activity against five reference fungal strains. Minimal inhibitory concentrations (MICs) determined by the microplate serial dilution method are shown in Table 2. To establish the possible mode of action we also analyzed the killing activity and determined the minimal fungicidal concentrations (MFCs) for selected derivatives with antifungal activity (Table 3).

**Table 2:** Susceptibility of fungal strains to analyzed anticancer drugs. Minimal inhibitory concentration (MIC) is defined as a concentration of a compound at which fungal growth is inhibited in 90% (MIC). No observable activity of a compound at measured concentrations is represented by (> 64). In this assay, the MIC value of fluconazole and amphotericin B were recorded as positive controls. For some compounds, higher concentrations were used to determine MIC (in rounded brackets). The experiments were performed in triplicates.

Compound	MIC µg mL^− 1^				
	*C. albicans* ATCC 10231	*C. glabrata* DSM 11226	*C. krusei* DSM 6128	*C. parapsilosis *DSM 5784	*S. cerevisiae* ATCC 9763
Idarubicin	64	1	4	2	2
Epirubicin	> 64	32	> 64	> 64	> 64
Daunorubicin	> 64	64	64	> 64	64
Doxorubicin	> 64	> 64	> 64	> 64	> 64
Valrubicin	> 64	> 64	> 64	> 64	> 64
Mitoxantrone	> 64 (256)	32	64	32	2
Pixantrone	> 64 (> 256)	32	64	32	32
Etoposide	> 64	> 64	> 64	> 64	> 64
Teniposide	> 64	> 64	> 64	> 64	> 64
Quinacrine	> 64	> 64	> 64	> 64	64
Bisantrene	> 64	> 64	> 64	> 64	4
Fluconazole	8	32	> 64	> 64	4
Amphotericin B	0.5	1	1	1	0.5

Although almost all analyzed compounds were strong yeast TopoII inhibitors (Table 1), only a few exhibited antifungal activity (Table 2). The most active against analyzed fungal strains appeared to be idarubicin and anthracenediones: mitoxantrone and pixantrone. Among anthracyclines (daunorubicin, idarubicin, epirubicin), high inhibitory potential and strong antifungal activity was observed only for idarubicin. It can be concluded that there is no simple correlation between enzyme inhibitory and antifungal activity.

Additional experiments performed for determining minimum fungicidal concentrations (MFCs) revealed fungicidal mode of action for the most active compounds (Table 3).

**Table 3:** Minimum fungicidal concentrations (MFC) determined for selected compounds. MFC is the lowest concentration resulting in the death of 99% of fungal cells. * > means no activity at the concentration mentioned. In this assay, the MFC values of amphotericin B were recorded as positive control. The experiments were performed at least in five replicates.

Compound	MFC^*^ µg mL^− 1^				
	*C. albicans* ATCC 10231	*C. glabrata* DSM 11226	*C. krusei* DSM 6128	*C. parapsilosis* DSM 5784	*S. cerevisiae* ATCC 9763
Idarubicin	> 256	4	32	16	2
Mitoxantrone	> 256	> 256	64	32	2
Pixantrone	128	> 256	128	32	32
Amphotericin B	2	2	2	2	2

Idarubicin was found to be the most active against all five reference fungal strains (Table 2), which was consistent with the highest yeast TopoII inhibitory effectiveness (Table 1). Interestingly, although idarubicin differs from daunorubicin only by the lack of 4-methoxy- group (Fig. 1) and both compounds inhibited yeast TopoII to a comparable extent, idarubicin’s antifungal activity significantly exceeded that of daunorubicin.

**Fig. 3:**
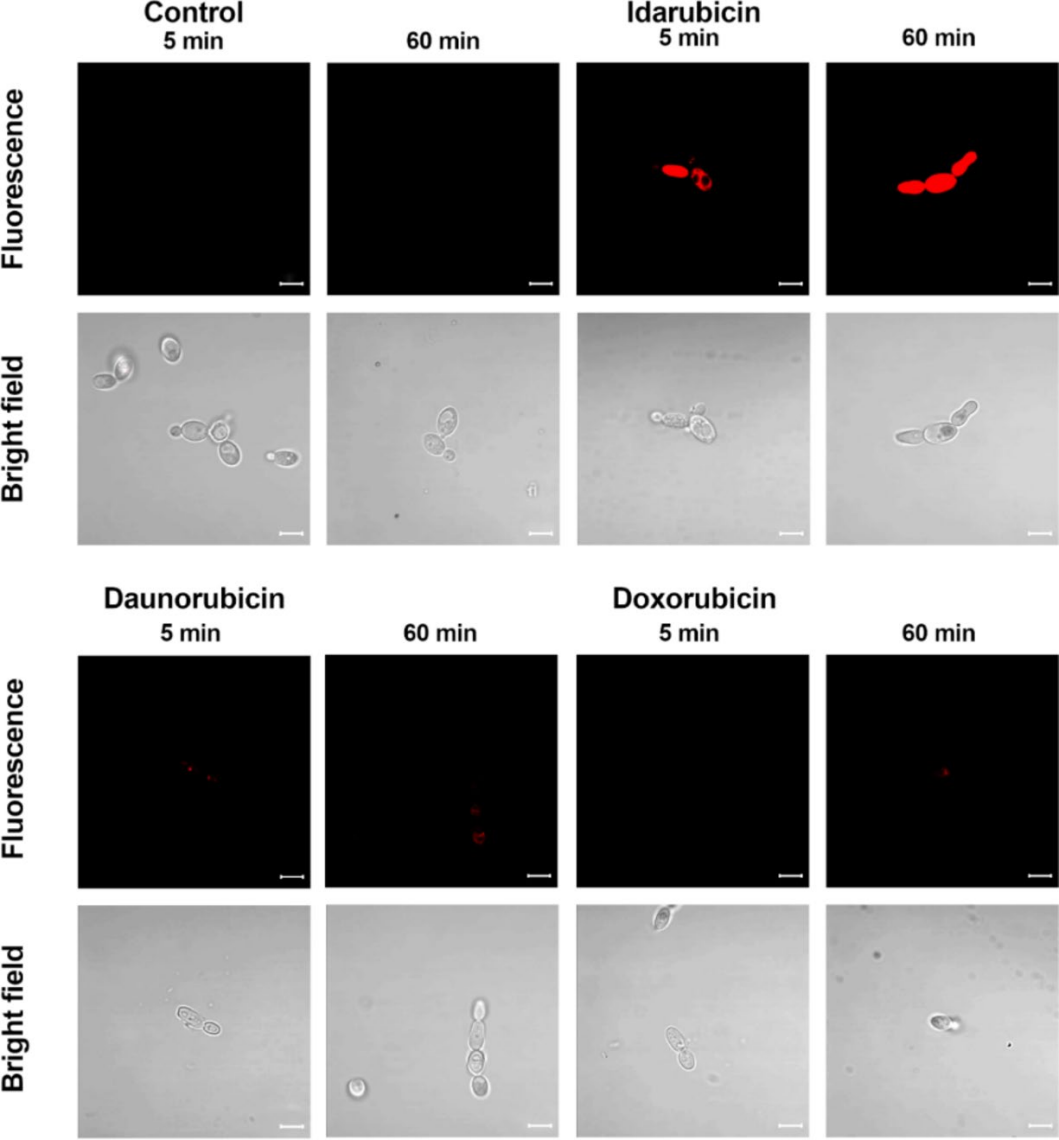
Fluorescence microscopic analysis of uptake and accumulation of idarubicin; daunorubicin and doxorubicin in *S. cerevisiae* cells ATCC 9763. Cells were suspended in phosphate-buffered saline and incubated in the presence of fluorescent probes at 100 µM of compounds concentration for an appropriate period of time. Scale bars correspond to 5 μm.

The fluorescent properties of anthracyclines^43^, characterized by a typical red fluorescence emission associated with their anthraquinone backbone, allowed us to perform flow cytometry analysis to better understand the basics of this effect. *S. cerevisiae* ATCC 9763 cells were treated with idarubicin, doxorubicin and daunorubicin at two time points. Results confirmed an increased population of idarubicin-positive cells and indicated an accumulation effect of this compound into fungal cells (Figure S3). For daunorubicin an accumulation effect, changing with time, was also noted, but at a much lower level. No accumulation was detected for doxorubicin. Results were consistent with decreasing antifungal activity of that three compounds mentioned (determined for *S. cerevisiae* MIC ^idarubicin^ 2 µg mL^− 1^ ≥ MIC ^daunrubicin^ 64 µg mL^− 1^> MIC ^doxorubicin^ above 64 µg mL^− 1^). Differences in uptake and accumulation of idarubicin, daunorubicin, and doxorubicin were also observed by fluorescence microscopy (Fig. 3). Results confirmed an efficient accumulation of idarubicin into fungal cells in contrast to daunorubicin and doxorubicin.

The observed limited penetration of anthracyclines into fungal cells prompted us to include valrubicin in the study group of compounds. That anthracycline derivative is anticipated to exhibit increased lipophilicity and enhanced capacity for rapid cellular penetration into human cells^36,37^. In a case of antifungal activity that compound seemed not to be transported into the fungal cells, metabolized into valerate-free metabolite, which was reported to be hTopoIIα poison^38^. Thus, no antifungal effect was observed (Table 2).

### Overcoming fluconazole resistance

*C. glabrata* clinical strains, fluconazole-resistant^44^, were used to analyze the possibility of overcoming fungal resistance by the use of the most active against *C. glabrata* DSM 11226 compounds (Table 2). MICs of the selected compounds against fluconazole-resistant *C. glabrata* clinical strains are presented in Table 4.

**Table 4:** Antifungal activity of selected compounds against fluconazole-resistant *C. glabrata* clinical strains in comparison with *C. glabrata* DSM 11226. * > means no activity at the concentration mentioned. In this assay, the MIC value of fluconazole was recorded as positive control. The experiments were performed at least in five replicates.

Strains	MIC^*^ µg mL^− 1^						
	Idarubicin	Epirubicin	Daunorubicin	Mitoxantrone	Pixantrone	Bisantrene	Flu
DSM 11226	1	32	64	32	32	> 64	32
CZD373	8	> 64	> 64	32	32	> 64	> 64
CZD377	32	> 64	> 64	32	32	> 64	> 64
CZD513	16	> 64	> 64	32	32	> 64	> 64
Gd 10	32	> 64	> 64	32	32	> 64	> 64

Although all analyzed in Table 4 compounds exhibited antifungal activity against fungal reference *C. glabrata* strain (Table 2) only idarubicin, mitoxantrone, and pixantrone were active against fluconazole-resistant clinical isolates. Moreover, idarubicin was the most effective.

Additional experiments were conducted to determine whether the highest antifungal activity of idarubicin against fluconazole-resistant clinical strains (Table 4) might be associated with the inability to efflux this drug by multidrug-resistance pumps. Thus, we used *S. cerevisiae* AD mutants, one from which seven major transporters of the ATP-binding cassette (ABC) family have been deleted (AD1-8u(-)) and mutant strains overexpressing genes encoding *C. albicans CDR1*, *CDR2*, and *MDR1* drug-efflux pumps^45,46^. MICs were determined in different conditions than used by us previously for reference strains, as indicated in the literature^45,46^. Mutant strains overexpressing genes encoding *C. albicans* drug-efflux pumps (*CDR1*, *CDR2*, and *MDR1*) became resistant to fluconazole in rich YPG (yeast extract, peptone, glucose) medium whereas *S. cerevisiae* AD1-8u (-) is hypersensitive to fluconazole regardless of the medium used^47^ (Table 5).

**Table 5:** MIC determined for *S. cerevisiae* strains in yeast synthetic Drop-out medium supplemented with uracil after 48 h of incubation and YPG (in rounded brackets). Reference ATCC 9763 strain was used for comparison.

S. cerevisiae	MIC^*^ µg mL^− 1^					
	Idarubicin	Daunorubicin	Mitoxantrone	Pixantrone	Bisantrene	Fluconazole
ATCC 9763	32 (64)	> 64 (> 64)	4 (64)	32 (64)	64 (> 64)	16 (32)
AD1-8u(-)	2 (2)	8 (8)	8 (64)	16 (32)	0.5 (0.5)	2 (2)
*Ca*Mdr1-GFP	8 (4)	8 (8)	16 (64)	8 (32)	2 (0.5)	> 64 (64)
AD*Ca*CDR1-GFP	4 (64)	16(> 64)	16 (64)	32(32)	2 (> 64)	4 (> 64)
AD*Ca*CDR2-GFP	2 (> 64)	8 (> 64)	8 (64)	16 (32)	0.5 (> 64)	2 (64)

For all analyzed drugs strong antifungal activity against *S. cerevisiae* AD1-8u(-) mutant strain, deficient in seven drug-efflux pumps, was observed in a minimal medium and except for mitoxantrone and pixantrone in rich YPG medium. As it can be seen in Table 5*Ca*Cdr1p, as well as *Ca*Cdr2p were able to use idarubicin, daunorubicin and bisantrene as substrates in contrast to *Ca*Mdr1 efflux-pump. Thus, *S. cerevisiae Ca*Mdr1-GFP strain remained sensitive and AD*Ca*CDR1-GFP as well as AD*Ca*CDR2-GFP became resistant to that compounds. Mitoxantrone and pixantrone with the lowest antifungal activity seemed not to be transported by analyzed types of efflux-pumps. Our results concerning Cdr1p, Cdr2p, and Mdr1p derived from *C. albicans* may also partly explain the highest MIC determined for idarubicin in a case of *C. albicans* ATCC 10231, significantly different from MICs observed for other fungal strains (Table 2).

### Yeast cells dysfunction upon idarubicin treatment

**Fig. 4:**
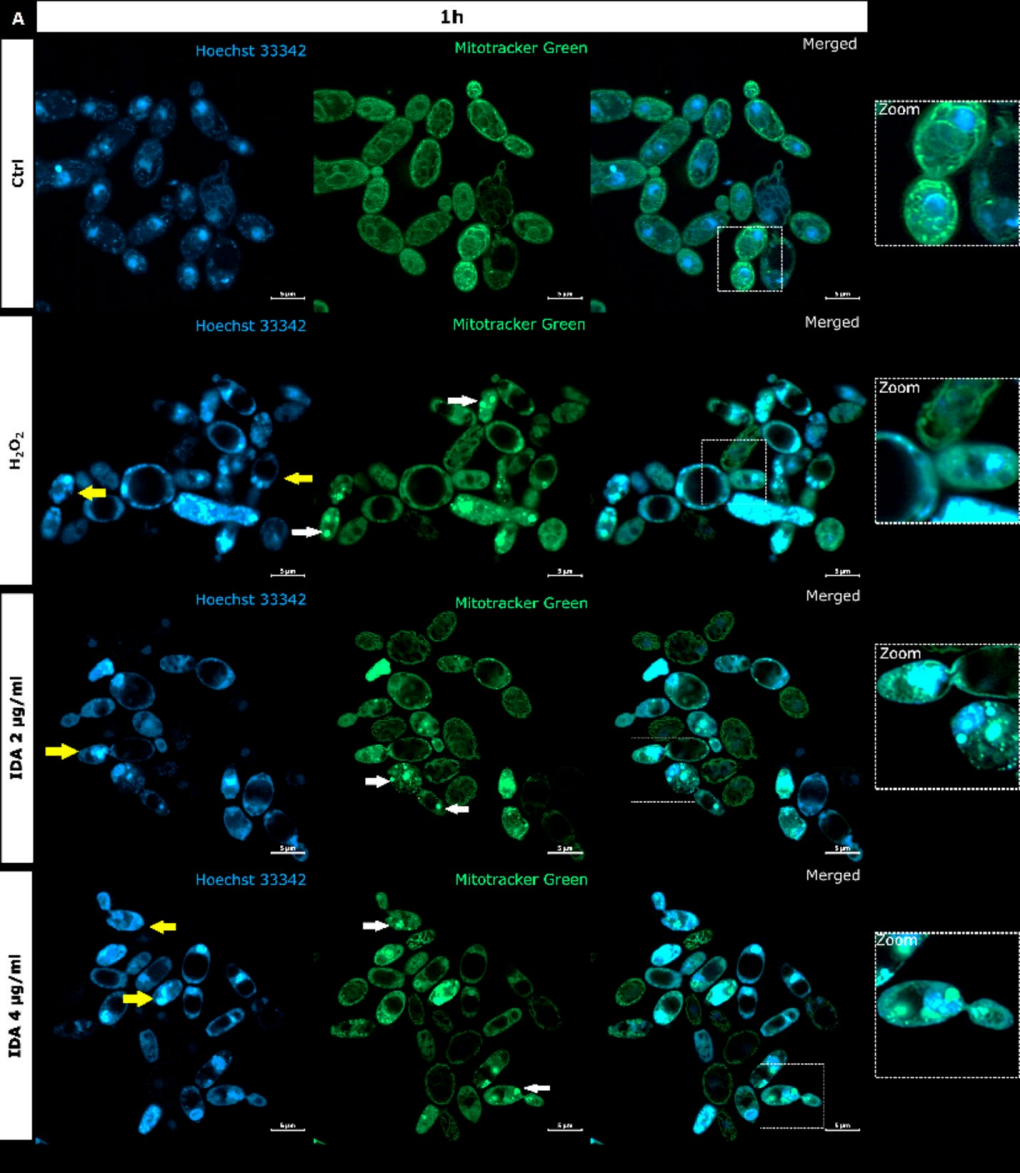

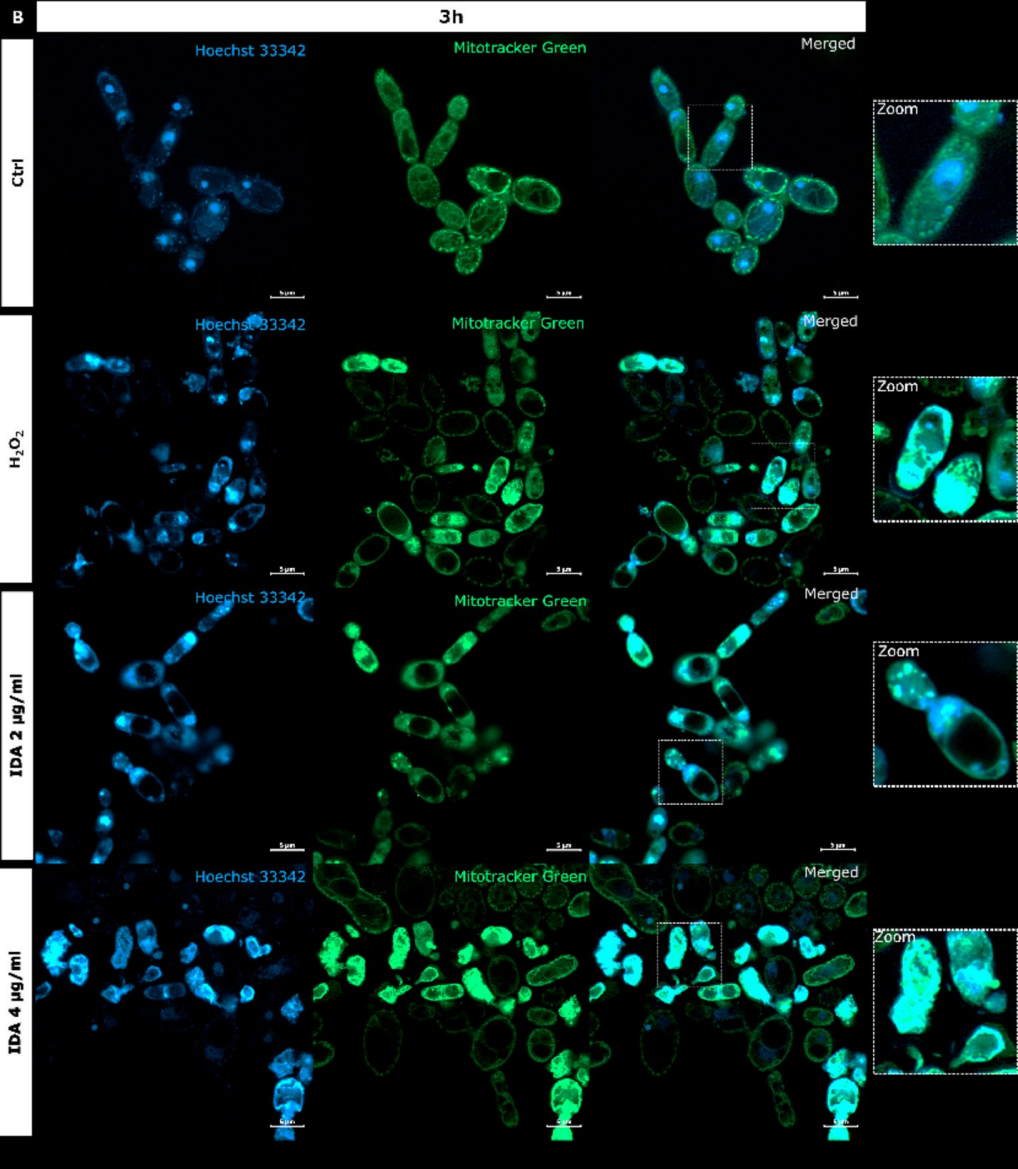
*S. cerevisiae* ATCC 9763 cells incubated for 1 h (**A**) or 3 h (**B**) with idarubicin 2 µg mL^− 1^, 4 µg mL^− 1^ and 100 µM H_2_O_2_ after staining with Hoechst 33342 and Mitotracker green. (**A**) White arrows indicate aggregated mitochondria, selected cells with fragmented genetic material undergoing the apoptotic process is marked by yellow arrows Under magnification in square frames, the fusion of mitochondrial DNA with nuclear DNA is visible. Scale bars correspond to 5 μm. Controls are indicated as Ctrl.

It is generally recognized that anthracyclines as anticancers act through a combination of multiple mechanisms and that the most consistent are, namely: intercalation into DNA, and more importantly, poisoning of hTopoIIα^48^. Because we proved the inhibitory activity of idarubicin against yTopoII, microscopic analysis was performed to observe for the first time the effect of idarubicin on *S. cerevisiae* cells morphology and yeast DNA topology, both nuclear and mitochondrial (mtDNA) (Fig. 4).

Our analysis revealed that upon staining with Hoechst 33342 in the presence of idarubicin nuclear DNA fragmentation was observed (marked with yellow arrows in Fig. 4A). The drug also affected the shape and cellular distribution of the mitochondrial network as observed upon staining with Mitotracker green (marked with white arrows in Fig. 4A). In yeast, mitochondria form a connected, tubular network that is evenly distributed in the cell, as can be seen in controls (Fig. 4). After treatment with idarubicin *S. cerevisiae* mitochondrial DNA (mtDNA) was targeted, leading to disruption of mitochondrial network architecture and mtDNA aggregation. Moreover, the fusion of mitochondrial DNA with nuclear DNA was also observed. After 3 h of incubation in 4 µg mL^− 1^ idarubicin concentration it was even not possible to distinguish nuclear as well as mitochondrial DNA (Fig. 4B). On the other hand, it was also reported that for mammalian cells idarubicin mechanism of action includes oxidative stress induction^49^. Thus, *S. cerevisiae* cells were treated by hydrogen peroxide to observe if the effect on yeast DNA (nuclear and mitochondrial) may be similar to that observed for idarubicin. In our hands mitochondria of *S. cerevisiae* cells were also targeted for oxidative damage after exposure to hydrogen peroxide, probably mediated by hydroxyl radicals generated by the Fenton reaction^50^. Mitotracker green staining indicated the presence of aggregated mitochondria in fungal cells treated with idarubicin as well as hydrogen peroxide (Fig. 4). Thus, our results confirmed that for fungal cells oxidative stress induction may also play a crucial role in idarubicin antifungal activity.

Similarly to hydrogen peroxide, idarubicin oxidative stress resulted in the presence of large and distinct vacuoles inside the yeast cells. In addition to its storage functions, vacuoles also serve an important detoxification function, through which multiple toxic molecules are sequestered away from the cytosol and other organelles^51^. It is thus probable, that idarubicin induced toxic metabolites were produced and fungal cells try to protect themselves from their harmful effects by removing them into the vacuole.

**Fig. 5:**
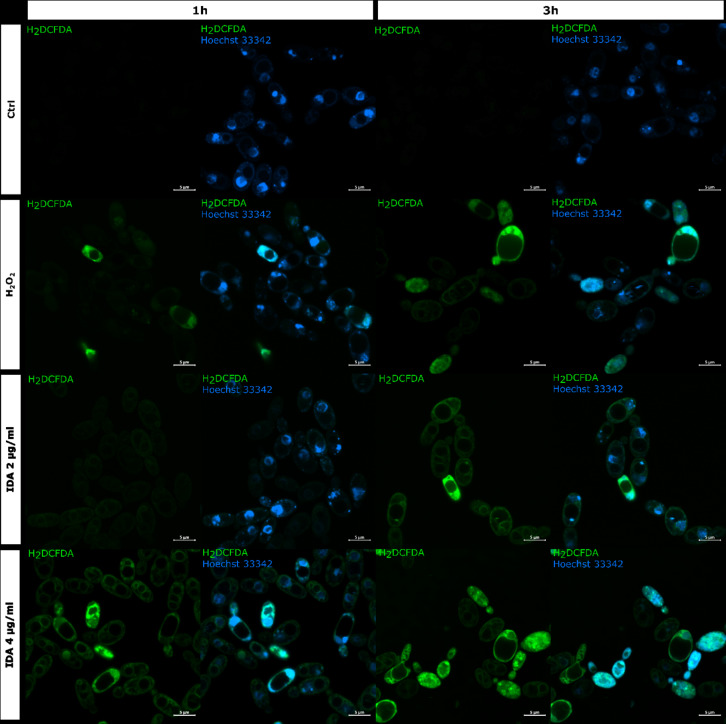
Detection of ROS in *S. cerevisiae* ATCC 9763 cells incubated for 1 and 3 h with idarubicin 2 µg mL^− 1^, 4 µg mL^− 1^ and 100 µM H_2_O_2_. Fluorescence images were obtained after staining with 2′,7′-dichlorofluorescin diacetate (H2DCF-DA) and Hoechst 33342. Controls are indicated as Ctrl. Bars represent 5 μm.

**Fig. 6:**
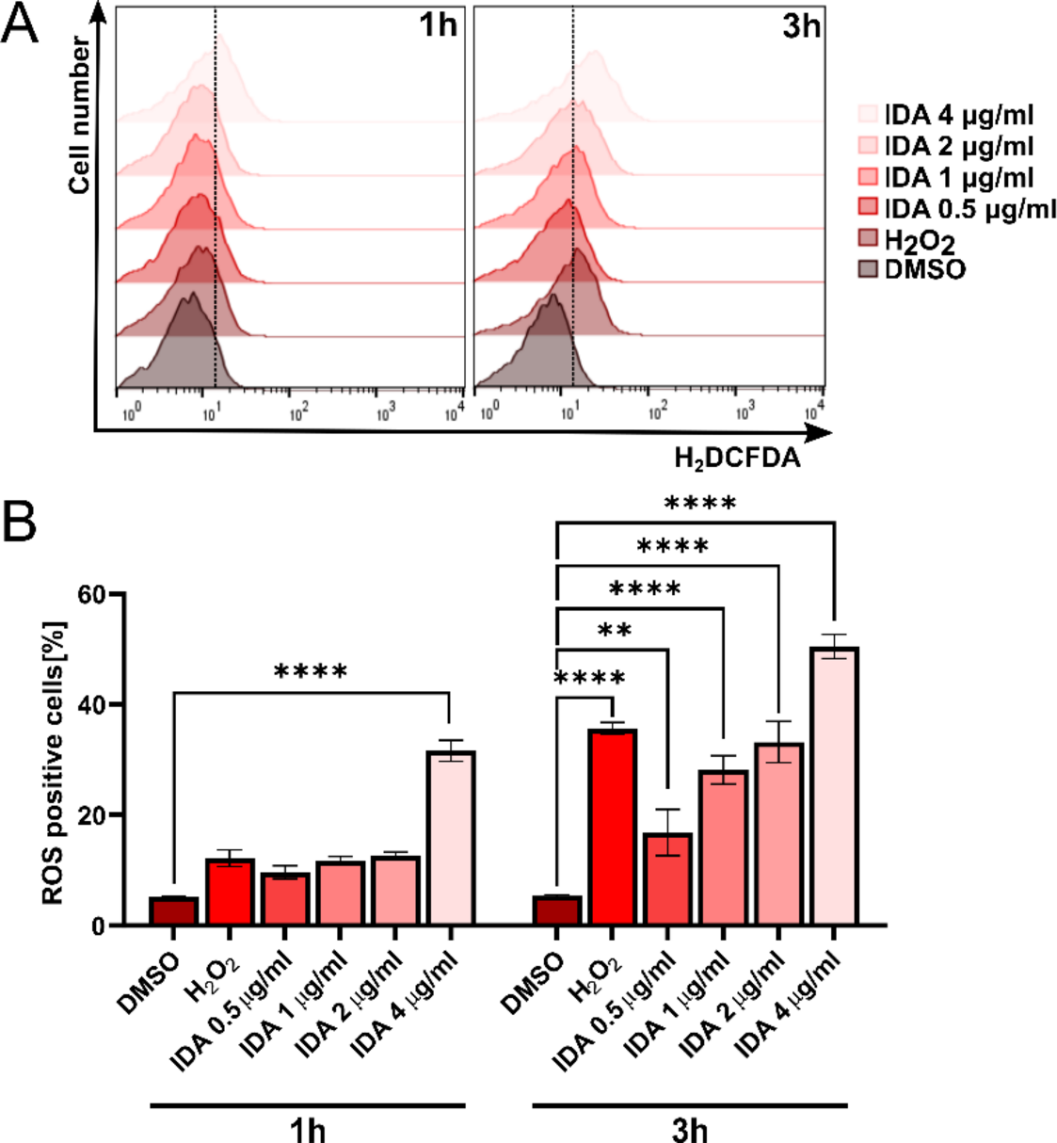
Assessment of ROS generation in *Saccharomyces cerevisiae* following idarubicin treatment via flow cytometry. DMSO and H_2_O_2_ were used as negative and positive controls, respectively. (**A**) Representative histograms demonstrate ROS induction across different treatment conditions; (**B**) Quantitative analysis of ROS levels. Error bars represent the SEM from three independent experiments (*n* = 3). Statistical significance is indicated as **p < 0.001, ****p < 0.00001 compared to the vehicle control (two-way ANOVA with Dunnett’s post hoc test).

To further confirm the formation of reactive oxygen species (ROS) in the presence of idarubicin additional microscopic experiments with 2′,7′-dichlorofluorescin diacetate (H2DCF-DA) staining as well as flow cytometry analysis were performed (Figs. 5 and 6).

Staining with 2′,7′-dichlorofluorescin diacetate (H2DCF-DA) is regarded as a rapid and sensitive assay for the detection of ROS. The oxidized form of the dye (DCF) is highly fluorescent, and visible as a green color localized in the cytoplasm of the yeast cell. The representative results of oxidative stress induction in the *S. cerevisiae* cells in the response of idarubicin as well as H_2_O_2_ are shown in Fig. 5. The presented effects were observed after an hour and 3 h of incubation of fungal cells with analyzed compounds. The control *S. cerevisiae* cells showed no fluorescence, which means that the polar form of H2DCF has not been oxidized to the fluorescent one. After the action of idarubicin at each concentrations, single cells or small groups of cells with clear green fluorescence located in the cytoplasm were detected. The intracellular fluorescence of the cytoplasm was also clearly differentiating from the unstained vacuoles. Observed by microscopic studies fluorescence intensity, indicating intense oxidative stress, was time and concentration-dependent.

Additionally, a flow cytometric analysis was performed to quantitatively assess the levels of reactive oxygen species (ROS) generated by *S. cerevisiae* cells following treatment with idarubicin at varying concentrations (0.5–4 µg mL^− 1^). As illustrated in Fig. 6, idarubicin effectively induces ROS production as early as 1 h post-treatment, with the effect intensifying over time and with increasing concentrations of the compound. Notably, even at low sub-MIC concentrations (0.5 µg mL^− 1^), idarubicin triggered a significant oxidative stress response, with statistical analysis confirming the significance (*p* = 0.0031).

This finding is particularly important, as it suggested that idarubicin’s ability to induce oxidative stress is evident at early stages, even before any effects on cell growth would typically be observed after prolonged exposure. The rapid increase in ROS levels, even at sub-MIC concentrations, indicated that it was one of the primary and immediate responses of the cells to idarubicin treatment. Furthermore, at the highest concentration tested (4 µg mL^− 1^), idarubicin induced higher ROS levels than the positive control, hydrogen peroxide (H₂O₂), highlighting its significant pro-oxidant activity. These results provided valuable insights into the early molecular effects of idarubicin in *S. cerevisiae* and suggested that oxidative stress may play a key role in the cellular response to the compound.

Taking into account all presented above examinations, idarubicin’s antifungal activity may be associated with apoptosis but it cannot be conclusively confirmed without further evidence. Apoptotic *S. cerevisiae* cells death mechanism involves the accumulation of reactive oxygen species (ROS), nuclear DNA fragmentation, chromatin condensation and mitochondrial dysfunction^52^.

### Influence on *C. albicans* virulence factors

**Fig. 7:**
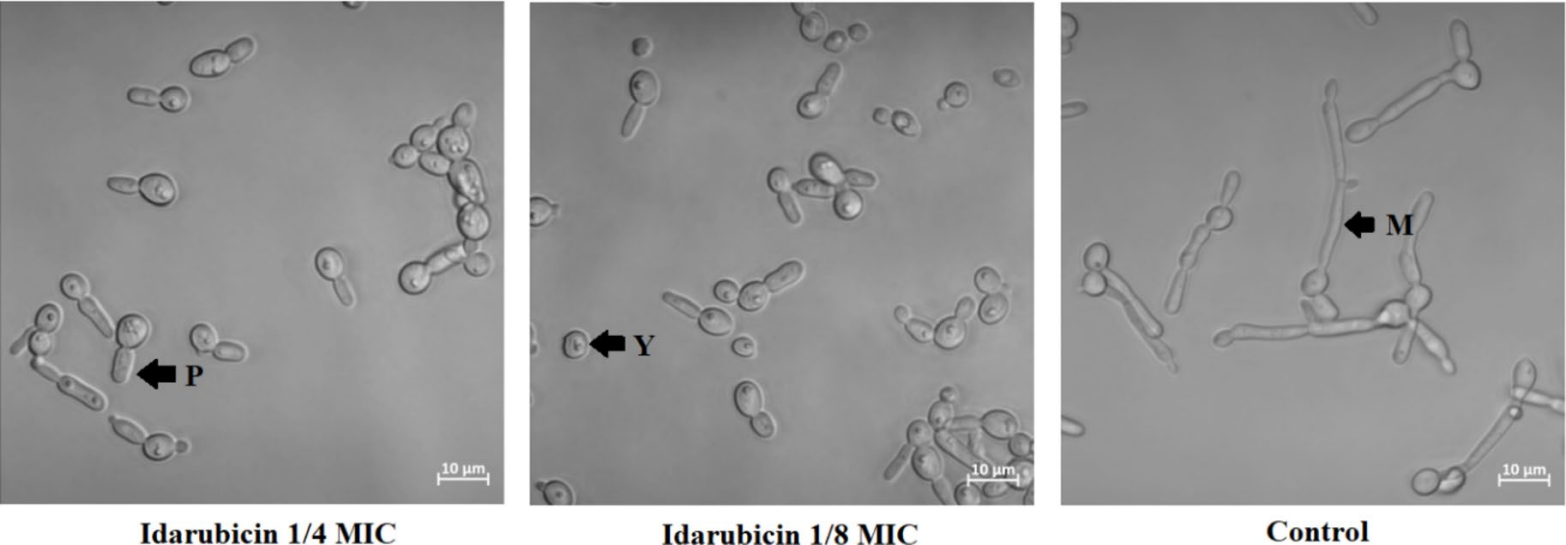
Microscopy analysis of changes in the morphology of *C. albicans* ATCC 10231 cells following treatment with idarubicin at 1/4 and 1/8 MICs. The cells were subjected to light microscopy, and the scale bar represents 10 μm. The analysis was conducted after a 3-hour incubation period at 37 ◦C in RPMI + 10% FBS medium. The experiments were performed at least in three replicates. P – pseudohyphae, Y – yeast and M – mycelial form.

*Candida albicans* is a dimorphic pathogenic yeast capable of producing alternate morphological forms (yeast or mycelium) in response to environmental changes. The ability of fungal cells to perform yeast-to-mycelium transformation as well as biofilm formation are important factors for virulence^53,54^. We have decided to analyze the effect of two compounds on *C. albicans* virulence factors due to the fact that only those two TopoII inhibitors exhibited antifungal activity against that particular strain. To check whether the presence of compound affects *C. albicans* ATCC 10231 morphological conversion, fungal cells were incubated under conditions stimulating this process in the presence of idarubicin and mitoxantrone at 1/4 and 1/8 MICs. To examine the inhibitory effects of the compounds against *C. albicans* biofilms, we used a microtiter-plate biofilm model. Both methods were previously described^23^. Microscopic analysis showed that the hyphal growth of *C. albicans* ATCC 10231 was predominantly inhibited in the presence of idarubicin at both concentrations (Fig. 7).

The presence of the compound did not cause the cells to retain at their yeast form, but induced the formation of pseudohyphae forms. In contrast to idarubicin, for mitoxantrone no effect on morphological transformation was observed (data not shown). The inhibitory effects of idarubicin and mitoxantrone against *C. albicans* biofilms was analyzed microscopically after incubation of fungal cells in the presence of compounds at the conditions stimulating biofilm formation. Microscopic analysis showed that idarubicin inhibited *C. albicans* ATCC 10231 biofilm formation and that this effect was concentration dependent (Figure S4). Thus, our results indicated that in the presence of clinically used idarubicin some candidal virulence factors might be strongly affected.

Summarizing, in order to be effective as antifungal, a drug needs to achieve plasma concentrations that are within therapeutic levels. In the case of idarubicin, the maximum plasma levels are lower than MICs^55^. Therefore, unlikely to be used as a therapeutic antifungal agent. However, the plasma concentration of idarubicin can substantially be increased by liposome encapsulation. Measured plasma levels of liposomal idarubicin reported previously^56,57^ is higher than MICs determined by us. Thus, such concentrations are sufficient for killing fungal cells. In addition, liposomal formulation usually improves the efficacy of a drug and reduces its toxicity at the same time^58^. Based on these findings, idarubicin might be further investigated as a possible new antifungal agent.

## Conclusions

Fungal infections are a serious threat to public health as they are becoming increasingly frequent and due to a rising fungal resistance to currently available antifungals. Therefore novel therapies are highly desirable. We proved that known topoisomerase II inhibitors, previously prescribed to treat non-infectious conditions, may display antifungal properties. Although both analyzed by us topoisomerases (yeast and human) originate from eukaryotic sources, moderate differences in sensitivity to idarubicin, epirubicin, and bisantrene were observed, interestingly with slightly greater inhibitory activity noted against fungal enzyme.

Clinical application of the most active idarubicin may be limited to treat life-threatening fungi, due to its toxicity, as anticancer drugs generally target eukaryotic organisms. On the other hand, affecting *C. albicans* virulence factors, such as morphological transformation and biofilm formation, is great advantage of idarubicin. The demonstrated fungicidal mode of action of idarubicin, mitoxantrone, and pixantrone is also their undoubted advantage due to the fact that killing activity is associated with a higher probability of early therapeutic success and a decreased probability of persistent or recurrent infection and resistance development. Additionally, analyzed compounds appeared to be active against fluconazole-resistant strains. Although, according to our results it is crucial to precisely define the type of infection and molecular basis of resistance. *C. glabrata* fluconazole-resistant clinical strains appeared to be sensitive to idarubicin, whereas *S. cerevisiae* AD mutants, overexpressing genes encoding *C. albicans CDR1*,* CDR2*, and *MDR1* drug-efflux pumps, differ significantly in their sensitivity to analyzed compounds. *S. cerevisiae Ca*Mdr1-GFP strain remained sensitive and AD*Ca*CDR1-GFP as well as AD*Ca*CDR2-GFP became resistant to idarubicin, daunorubicin, and bisantrene.

Assuming that obstacles may be overcome, our results indicated that fungal topoisomerase II targeting is worth considering in antifungal treatment and that these reported drugs could serve as a starting point for the reinnovation of a new molecule.

## Materials and methods

### Strains

The fungal strains employed in scientific research: collection strains: *Candida albicans* ATCC 10231, *Candida glabrata* DSM 11226, *Candida krusei* DSM 6128, *Candida parapsilosis* DSM 5784 *and Saccharomyces cerevisiae* ATCC 9763, fluconazole-resistant *C. glabrata* clinical strains obtained courtesy of Piotr Szweda (Gdansk University of Technology): CZD373, CZD377, CZD513, Gd 10^44^ and *S. cerevisiae* mutant strains obtained courtesy of Rajendra Prasad (School of Life Sciences, Jawaharlal Nehru University, New Delhi, India ): AD1-8u(-), *Ca*Mdr1-GFP, AD*Ca*CDR1-GFP, AD*Ca*CDR2-GFP^45–47^. Yeast strains were cultured on solid and liquid YPG medium (1% m/V yeast extract; 1% m/V peptone; 2% m/V glucose; 1.5% m/V agar) for 18 h at 30 °C in a shaking incubator (INFORS HT Bottmingen, Switzerland).

### Determination of the minimum inhibitory concentration of a growth-inhibiting compound against fungi (MIC)

MIC parameters were determined according to the previously described method^24^. Serial dilutions of the tested compounds were performed in a 96-well microtiter plate using an appropriate medium. An inoculum of cells was added (10^4^ CFU). The plates were incubated for 18 h at 30 °C (48 h for *S. cerevisiae* mutants). Results were read at 600 nm using a TECAN Spark 10 M microplate reader.

RPMI-1640 (Sigma- Aldrich, St. Louis, MO, USA) medium buffered to pH 7.0 were used for all collection strains and clinical strains of *C. glabrata* while *S. cerevisiae* mutants were grown in ) YPG medium or 0.67% m/V YNB medium (MP Biomedicals, Irvine, CA, USA) without amino acids, folic acid, p-aminobenzoic acid, with ammonium sulfate supplemented with 2% m/V glucose, 0.192% m/V yeast synthetic drop-out medium supplement without uracil (Sigma-Aldrich, St. Louis, MO, USA) and 0.0076% m/V uracil (Sigma-Aldrich, St. Louis, MO, USA.

### Determination of the minimum fungicidal concentration (MFC)

The MFC parameter was determined by spot assay using a microplate replicator and similarly prepared 96-well microtiter plates as used for MIC value determination. The MFC parameter was defined as the lowest compound concentration at which no recovery of microorganisms was observed.

### The effect of Idarubicin on *C. albicans* morphological conversion and biofilm formation

The effect of idarubicin on *Candida albicans* ATCC 10231 yeast-to-mycelium transformation as well as biofilm formation was analyzed with the use of a microtiter plate-based method according to the previously described protocols in RPMI-1640 + 10% (by volume) fetal bovine serum (FBS) medium^23^. Following the incubation period (3 h for morphological conversion and 18 h for biofilm formation), the cells were washed three times with PBS and imaged using an LSM 800 inverted laser-scanning confocal microscope from Carl Zeiss (ZEISS, Göttingen, Germany), equipped with a × 63 1.4 NA Plan Apochromat objective from Carl Zeiss (for imaging morphological conversion) or Tecan Spark 10 M Multimode Plate Reader equipped with bright field imaging system (4x objective, for biofilm analysis).

### Flow cytometry

An overnight culture of *S. cerevisiae* in YPG medium was washed twice with sterile phosphate-buffered saline (PBS) and diluted to an optical density of 1 at 600 nm. The inoculum was incubated at 30 °C with 4 µg mL^-1^ of the compounds for 5 and 60 min. After incubation, 1 mL of the inoculum was washed twice with PBS and the cell suspension was examined with the NovoCyte Flow Cytometer (Agilent, San Diego, CA, USA) using a blue laser with an excitation wavelength of 488 nm and an emission wavelength of 572 nm, and the data were analyzed using FlowJo v10.8.0 software (BD Life Sciences, Franklin Lakes, NJ, USA). Each experiment was independently conducted three times.

Assessment of ROS generation in *S. cerevisiae* cells by flow cytometry was performed similarly to the microscopic analysis, with the exception of idarubicin concentrations, which were 0.5, 1, 2, and 4 µg mL^-1^. Following treatment, cells were stained with 2′,7′-dichlorofluorescin diacetate (H2DCFDA) probe (C6827, Thermo Fisher Scientific) at 7 µM, added thirty minutes before the end of incubation. The cells were then centrifuged at 5000 rpm for 5 min, washed in PBS buffer, centrifuged again, and analyzed by flow cytometry using the Guava EasyCyte 8 Cell Sorter (Merck Millipore). Data were processed with software mentioned above. Each experiment was independently conducted three times.

### Staining and microscopic analysis

*Sacharomyces cerevisiae* ATCC 9763 was initially cultured on a standard YPG rich medium at 30 °C for 24 h. Subsequent experiments utilized YPG poor liquid medium, composed of 0.1% yeast extract, 0.2% glucose, and 0.05% peptone. Idarubicin was added at final concentrations of 2 µg mL^− 1^ and 4 µg mL^− 1^ to a medium containing the yeast culture at an optical density of 0.5 (OD_660_). Hydrogen peroxide (H₂O₂), at a concentration of 100 µM, served as the positive control. Samples were then incubated on a shaker at 30 °C for durations of 1 h and 3 h. Depending on the specific experiment, cells were stained with 2′,7′-dichlorofluorescin diacetate (H2DCFDA) probe (C6827, Thermo Fisher Scientific) at 7 µM, thirty minutes before the end of incubation to detect reactive oxygen species, or with MitoTracker Green (M7514, Thermo Fisher Scientific) at 100 nM, fifty minutes before the end of incubation to image mitochondria. In both cases, Hoechst 33,342 at 5 µg mL^− 1^ (14533, Sigma-Aldrich) was also added for nuclear visualization. After staining, cells were centrifuged at 5000 rpm for 5 min, washed in PBS buffer, and centrifuged again.

The inoculum for the compound accumulation test was prepared similarly to the flow cytometry test and diluted to an OD_600nm_ 0.8 − 0.7 (equivalent to 10^7^ CFU). The cells were incubated at 30 °C with 100 µM of the compounds for 5 and 60 min. After incubation, 1 mL of the inoculum was washed twice with PBS and resuspended in 80 µL of 90% glycerol (by volume) and 10% tenfold concentrated PBS (by volume).

All prepared samples were mounted on microscopic slides and analyzed using an LSM 800 inverted laser scanning confocal microscope (Carl Zeiss), equipped with an Airyscan detector and a ×63 1.4 NA Plan Apochromat objective. Imaging parameters such as laser intensity, exposure times, and gain settings were consistently maintained across all samples to ensure comparability.

### Yeast and human topoisomerase II relaxation assay and Inhibition

Relaxation assays were performed according to the Inspiralis protocols (Inspiralis Ltd., NR4 7GJ Norwich, UK). The reaction with human topoisomerase alpha II was carried out in Assay buffer containing 5 mM Tris-HCl (pH 7.5), 12.5 mM NaCl, 1 mM MgCl_2_, 0.5 mM DTT, 10 µg mL^− 1^ albumin, with the addition of 1 mM ATP, 250 ng of supercoiled pBR322, and the appropriate amount of the tested compounds. The reaction was initiated by adding the enzyme and conducted for 30 min at 37 °C. After this time, the reactions were stopped by adding 5 µL of Gel Loading Dye, Purple (6X) (New England, Biolabs, Ipswich, UK). A two-step extraction was performed, using water-saturated butanol to remove the upper (butanol) layer, followed by extraction with 30 µL of chloroform/isoamyl alcohol (by volume, 24:1). The upper layer, 5 µL, was applied to a 1% w/v agarose gel. Electrophoresis was carried out in 1× TAE buffer for 3 h at 4.5 V cm^− 1^. The gel was stained for 30 min in GelRed 3× staining solution and photographed with a Gel Doc XR + Gel Documentation System (Bio-Rad Laboratories, Inc., 1000 Alfred Nobel Drive, Hercules, CA, USA).

The relaxation assay conducted with yeast (from *S. cerevisiae*) topoisomerase II (without and with analyzed compounds) was performed similarly, but the composition of the assay buffer differed: 1 mM Tris-HCl (pH 7.9), 10 mM KCl, 0.5 mM MgCl₂, 0.2% (v/v) glycerol, and the reaction temperature was 30 °C.

The IC_50_ values were determined using GraphPad Prism 9 software as the concentration of the compound that inhibits 50% of the enzyme’s relaxation activity by densitometry quantification of the transition from supercoiled to relaxed forms and was expressed in relation to the control.

### Statistics and reproducibility

Experiments regarding the determination of the MIC and MFC parameters were performed at least in five replicates. All other experiments were conducted in at least three replicates. The means ± SD were used for statistical analysis of IC50. For quantitative analysis of ROS levels error bars represent the SEM from three independent experiments (*n* = 3). Statistical significance was determined in comparison to the DMSO-treated control group (1% v/v) using two-way ANOVA followed by Dunnett’s post hoc test. Statistical analysis was conducted using GraphPad Prism 9 software.

## Electronic supplementary material

Below is the link to the electronic supplementary material.

Supplementary Material 1

## Data Availability

Data generated or analyzed in this study are included in this published article and supplementary material files. Raw data supporting our results are also available as data sets: Kondaka, K., Rząd, K., & Gabriel, I. (2024). Effect of antitumor compounds on the yeast topoisomerase II relaxation activity (1–) [dataset]. Gdańsk University of Technology.https://doi.org/10.34808/zj53-xt83; Kondaka, K., Rząd, K., & Gabriel, I. (2024). Minimum inhibitory concentrations (MICs) determination of selected human topoisomerase II alpha and bacterial DNA gyrase inhibitors against fungal strains (1–) [dataset]. Gdańsk University of Technology. https://doi.org/10.34808/0kz4-y909.
