# Supplementary material for: Anticancer drugs targeting topoisomerase II for antifungal treatment
Source: Sci Rep. 2025 Mar 18;15:9311. doi: 10.1038/s41598-025-93863-z (PMC11920057; doi:10.1038/s41598-025-93863-z)
Supplement: Supplementary file 1 — Supplementary Material 1 [file 41598_2025_93863_MOESM1_ESM.pdf]

Anticancer drugs targeting topoisomerase II for antifungal treatment

Kavya Kondaka , Kamila Rząd, Natalia Maciejewska, Iwona Gabriel *

Department of Pharmaceutical Technology and Biochemistry, Faculty of Chemistry, Gdansk University of Technology, 11/12 Narutowicza Str., 80-233 Gdansk, Poland

*Correspondence: Tel: +48 583486078; E-mail: iwona.gabriel@pg.edu.pl

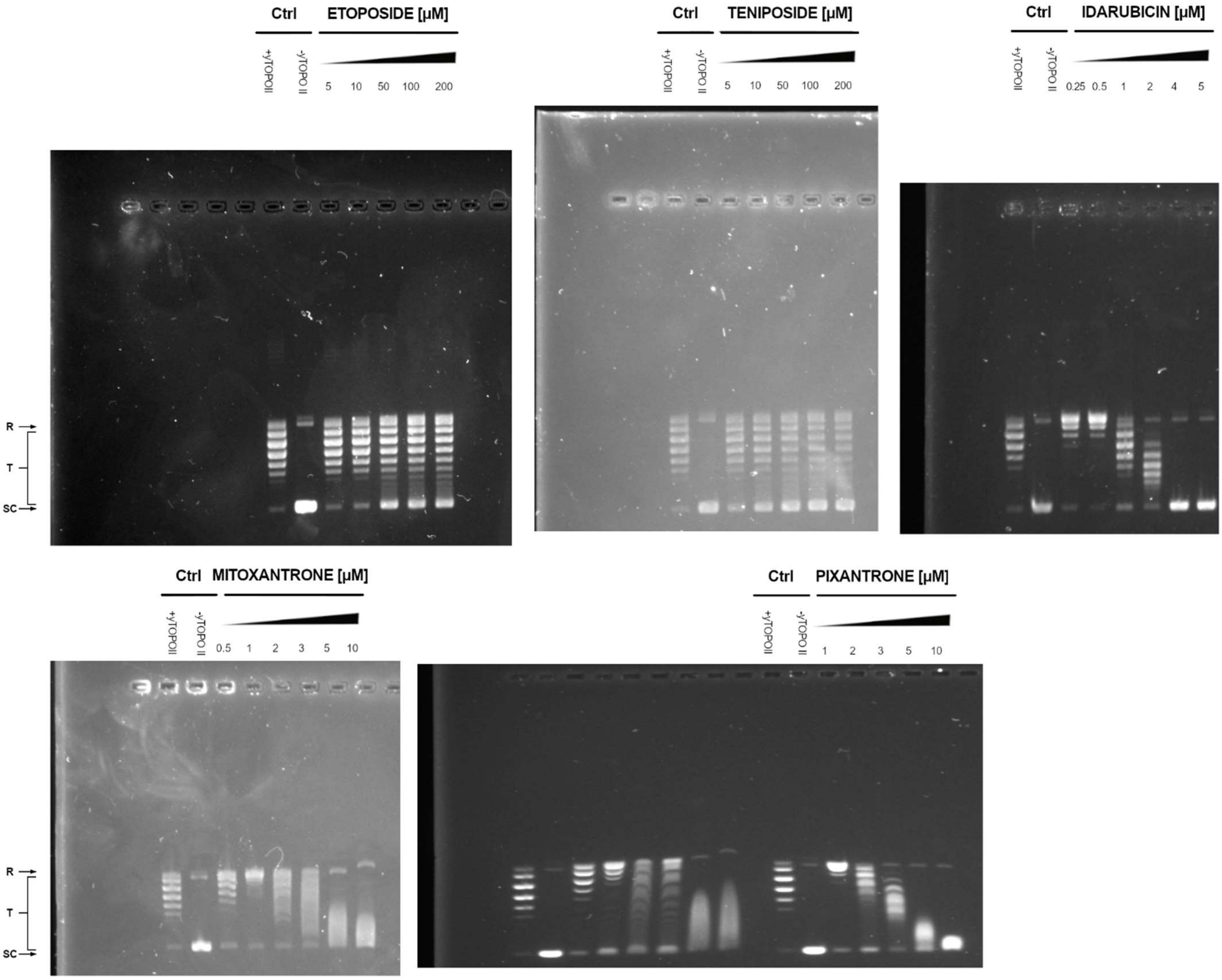

Figure S1 Inhibition of the relaxation activity of yeast topoisomerase II (Inspiralis) by selected compounds: etoposide, teniposide, idarubicin, mitoxantrone, and pixantrone. Purified yeast topoisomerase II was used to relax supercoiled pBR322 plasmid DNA, either in the absence (+yTOPO II) or presence of the compound being tested. SC stands for supercoiled DNA, R for relaxed DNA, and T for DNA topoisomers. The various topological forms of DNA were separated using a 1% agarose gel. The data presented are representative of three independent experiments.

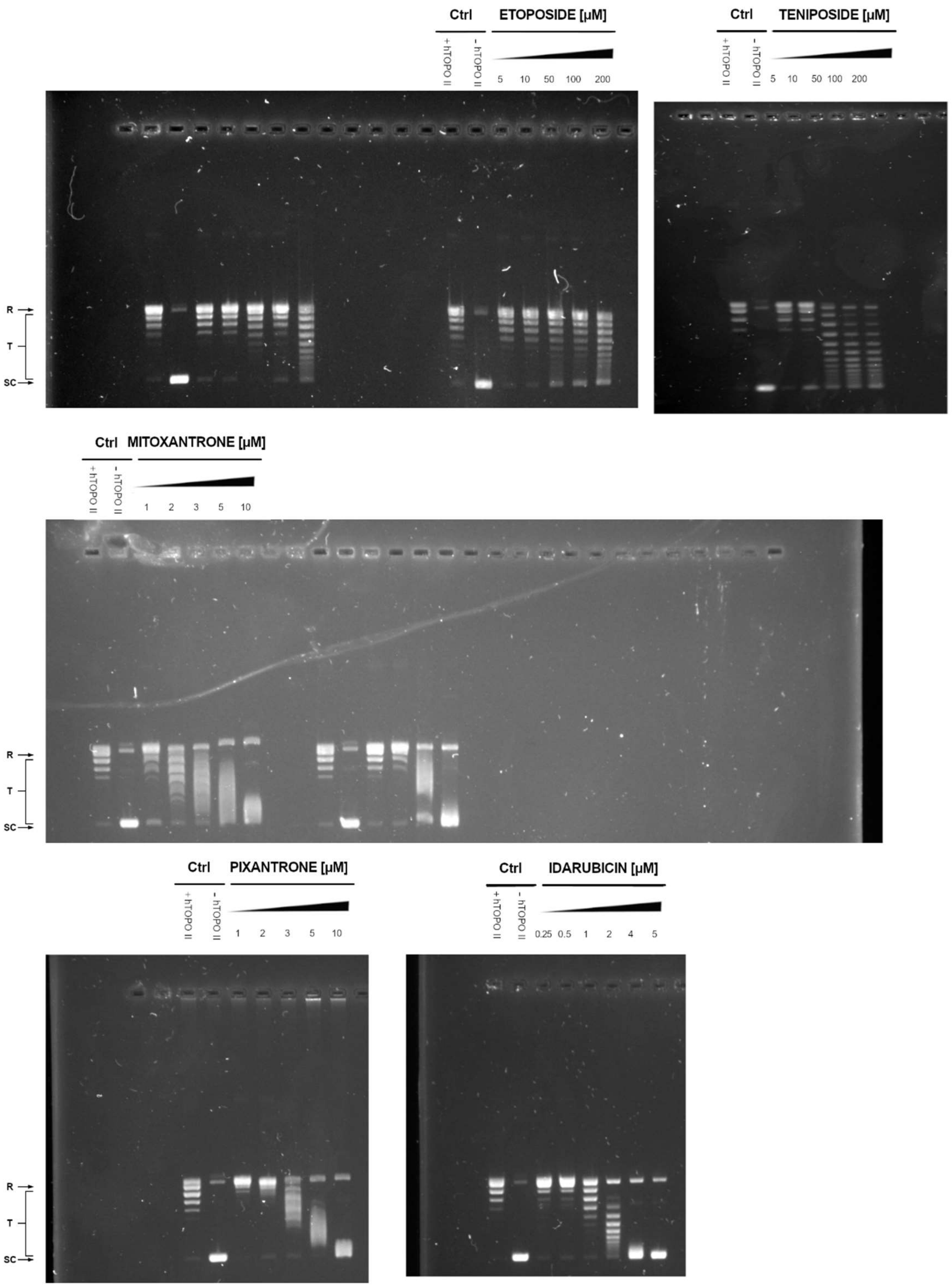

Figure S2 Inhibition of the relaxation activity of human topoisomerase II alpha (Inspiralis) by selected compounds: etoposide, teniposide, idarubicin, mitoxantrone, and pixantrone. Purified yeast topoisomerase II was used to relax supercoiled pBR322 plasmid DNA, either in the absence (+yTOPO II) or presence of the compound being tested. SC stands for supercoiled DNA, R for relaxed DNA, and T for DNA topoisomers. The various topological forms of DNA were separated using a 1% agarose gel. The data presented are representative of three independent experiments.

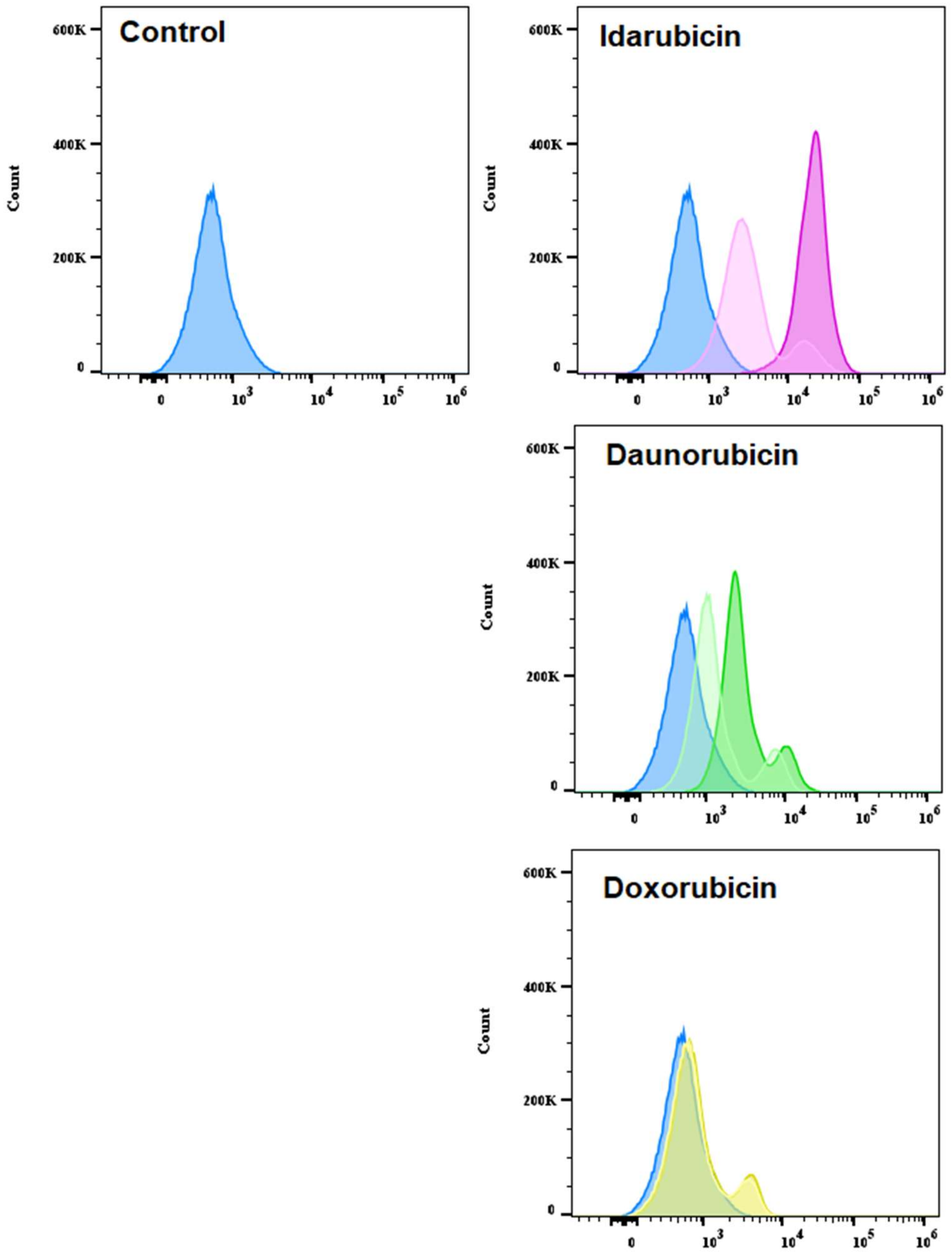

Figure S3. Representative histograms of flow cytometry analysis of fungal cells treated with idarubicin, daunorubicin and doxorubicin at the concentrations 4 µg mL^{-1}. Analysis was performed after 5 min (a lighter color palette) and 1h (a darker color palette) of incubation time with blue laser excitation wavelength 488 nm and emission 572 nm.

1/4 MIC **1/8 MIC**

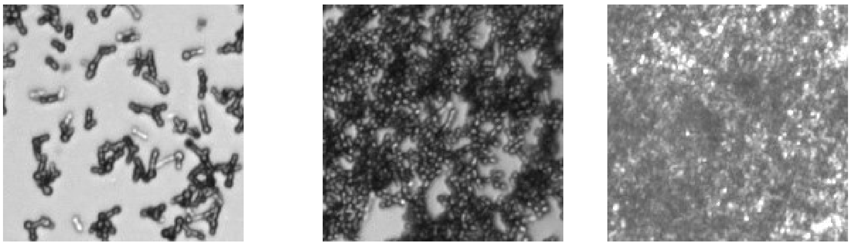

Idarubicin **Control**

Figure S4. Microscopy analysis of changes in the biofilm formation of *C. albicans* ATCC 10231 cells following treatment with idarubicin. The fungal biofilm imaging was performed with the use of Tecan Spark 10M Multimode Plate Reader equipped with bright field imaging system (4x objective). The analysis was conducted after a 18-hour incubation period in RPMI-1640 + 10% FBS at 37° C. The experiments were performed at least in three replicates.
